# Cryogenic Coaxial Printing for 3D Shell/Core Tissue Engineering Scaffold with Polymeric Shell and Drug-Loaded Core

**DOI:** 10.3390/polym14091722

**Published:** 2022-04-22

**Authors:** Tianqi Liu, Bo Yang, Wenqing Tian, Xianglin Zhang, Bin Wu

**Affiliations:** State Key Laboratory of Materials Processing and Die & Mould Technology, School of Materials Science and Engineering, Huazhong University of Science and Technology, Wuhan 430074, China; ltqfxclhl@foxmail.com (T.L.); m202071032@hust.edu.cn (B.Y.); m201970934@hust.edu.cn (W.T.); hust_zxl@mail.hust.edu.cn (X.Z.)

**Keywords:** additive manufacturing, polycaprolactone, cryogenic coaxial printing, responsive drug release

## Abstract

For decades, coaxial printing has been widely applied in 3D tissue engineering scaffold fabrication. However, there are few reports regarding polymeric materials application in shell production due to fabrication constraints. In this study, a combination of cryogenic printing and coaxial printing aims to approach the challenge. Polycaprolactone (PCL) and sodium alginate (SA) were selected as the representative shell and core materials to test the feasibility of the coaxial cryogenic printing by optimizing key parameters, including working temperature, air pressure, PCL, and SA concentration. According to the optical and SEM images, the SA core contracts a string inside the PCL shell, illustrating the shell/core structure of the 3D coaxial PCL/SA scaffolds. Besides, the shell/core 3D scaffold possesses a 38.39 MPa Young’s modulus in mechanical tests; the PCL shell could retain at least 8 h in 5 mol/L HCl solution, leading to a fabricated drug-loaded PCL/SA shell/core “responsive” to acidic pH. In summary, coaxial cryogenic printing was developed to fabricate 3D scaffolds with a PCL/SA shell/core scaffold, broadening the material range of coaxial printing and providing promising applications in drug release.

## 1. Introduction

Traditional 3D printing has been widely used in tissue engineering for tissue repair and regeneration, such as fused filament fabrication (FFF) [1], embedded printing [2], etc. Recently, a special type of 3D printing, coaxial printing, has been used in tissue engineering. Compared to the single nozzle used in traditional 3D printing, the nested coaxial nozzles provide more possibility and creativity. Firstly, a scaffold with hollow tubular fiber can be achieved through printing the precursor and crosslinker [3], sacrificial inner material [4], or viscous outer material [5]. The achieved hollow structure in the 3D scaffold was believed to accelerate angiogenesis [6]. Secondly, two different kinds of living cells can be encapsulated in the outer and inner material separately to realize the spatial location of multiple types of cells, mimicking the arrangement of the cells in natural organs [7]. Thirdly, different kinds of materials, such as polymers and hydrogels, can be fabricated into core/shell structures, providing different advantages. For example, when the polymeric material was used as the shell, it could adjust the drug release [8]; when the polymeric material was used as the core, it could be used to reinforce the mechanical property of the whole scaffold [9]. Especially, sodium bicarbonate was used as the shell material to provide a pH-sensitive drug delivery [10], and the feed ratio of the shell and core material was controlled to regulate the scaffold stiffness [11].

Even though the coaxial printed scaffold has some advantages, the biomaterials available for coaxial were restricted due to the high fabrication requirements. The existing coaxial printing normally uses hydrogel or ceramic; polymeric materials are often used to prepare scaffolds for tissue engineering repair [12] but are, reportedly, rarely used to fabricate 3D coaxial scaffolds. The reason is the fabrication limitations. Firstly, during the hydrogel coaxial printing, the precursor and crosslinker can be printed simultaneously through outer and inner nozzles to realize the in situ crosslinking. Alginate-based hydrogel and calcium ion are common material sets [13]. Secondly, the ceramics were normally prepared as viscous slurry, possessing self-set ability. Then, the ceramic slurry was extruded to form the green part, followed by sintering [14]. The sintering process could damage living cells or growth factors. Thirdly, the polymeric material is rarely fabricated into a 3D scaffold through coaxial printing. This kind of material was mostly fabricated into coaxial nano-wires through electrospinning [8,9,10], such as when Huihua Yuan studied the effect of biomimetic COL1-CS (shell)/PLLA (nucleus) fibers on the tendon differentiation of human mesenchymal stem cells (hMSCs), and Piyachat Chuysinuan developed a nanofibrous core–sheath structured scaffold comprising a tetracycline-loaded alginate/soy protein isolate (TCH-Alg/SPI) as a core and polycaprolactone (PCL) as a sheath using coaxial electrospinning. The solvent of nano-wires can be volatilized rapidly under high voltage. However, when the diameter of the fiber increases, the increased amount of solvent cannot be volatilized quickly, leading to the failure of the fiber formation and the collapse of the 3D scaffold.

Cryogenic printing was combined with coaxial printing to fabricate a 3D coaxial scaffold with a polymeric shell in this study. Relative to traditional hot-melt molded scaffolds, the cryogenic coaxial printed scaffolds obtained in this study have comparable mechanical properties [15]. Furthermore, the cryogenic printing condition provides extra advantages, such as a friendly temperature for living cells and growth factors, and nanopores on the fiber surface, which is beneficial for cell adhesion, proliferation, and differentiation as well as drug containing [16].

The cryogenic coaxial printed scaffold with a polymeric shell obtained in this study has promising applications in tissue engineering. It can be used as a responsive drug release scaffold aiming at infected tissue repair. Bacterial infection brings more difficulty and complexity to tissue defects regeneration [17]. For example, Staphylococcus-caused septic arthritis and osteomyelitis lead to graft failure, which is because the bacteria could damage the local vasculature and hamper the nutrition supply [18]. In order to overcome the damage caused by bacteria, researchers used physisorption [16], hydrogel encapsulation [19], and other methods to achieve drug delivery.

We utilized a “responsive drug release” strategy that releases drugs only focused on the infected area. Polycaprolactone (PCL) is a kind of protective sheath material. It can be degraded much faster in an acidic solution as well as lipase [20]. As a result, the PCL sheath can be dormant in the normal environment. In the infected area, the bacteria-caused [21] change in the pH [22] and enzyme can trigger the drug release, and this strategy was named “on-demand” drug delivery. This strategy overcomes the inability of drugs to maintain and “recognize” infected areas in the body for long periods of time due to methods such as physisorption and hydrogel encapsulation. Coaxial printing has been used to fabricate a single drug-loaded rod with a PCL sheath and alginate core to prolong the drug release time [23]. Based on these valuable results, we go a step further, fabricating a responsive drug release 3D scaffold with a PCL shell and drug-loaded alginate core.

## 2. Materials and Methods

### 2.1. Materials

Polycaprolactone (PCL) pellets (MW ~80,000) were purchased from Perstorp UK Limited (Warrington, UK), while glacial acetic acid (GAC) (purity > 99.5%) was obtained from Sinopharm Chemical Reagent Co., Ltd. (Shanghai, China). Alginate was purchased from Shanghai Aladdin Biochemical Technology Co., Ltd. (Shanghai, China). Levofloxacin (purity > 98%) was obtained from Shanghai Macklin Biochemical Co., Ltd. (Shanghai, China). Universal red ink was purchased from Shenzhen Caiju Electronic Technology Co., Ltd. (Shenzhen, China). All reagents were used in the experiments without further processing.

### 2.2. Cryogenic Coaxial Printing

The scaffolds made by cryogenic coaxial printing were named CCP scaffolds, while the cryogenic printing scaffolds were named CP scaffolds. The CCP scaffolds were made by the following steps.

**Figure 1:**
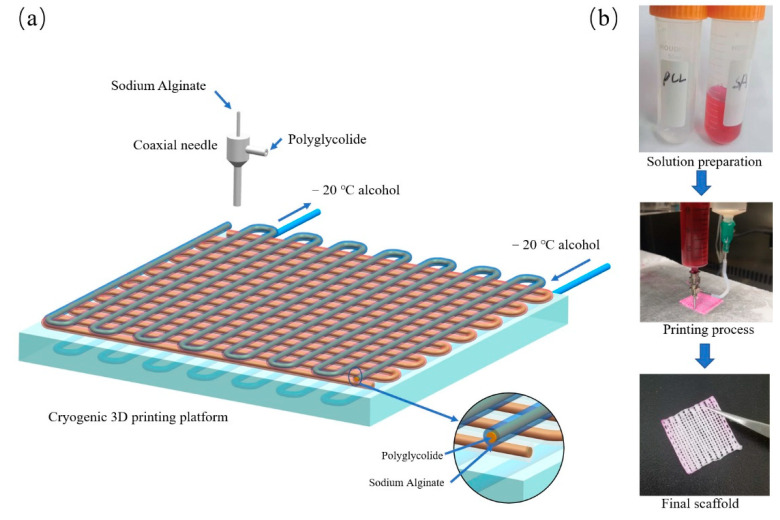
(**a**) The schematic diagram of cryogenic coaxial printing with PCL as the shell and SA as the core; (**b**) photographs of materials, process, and final 3D scaffold.

A 40 wt.% polycaprolactone (PCL) solution was prepared by dissolving PCL pellets in GAC through continuous magnetic stirring at 100 rpm for 6 h at 65 °C. The solution was then centrifuged at 2000 rpm for 2 min to remove any bubbles and subsequently loaded in a 10 mL plastic syringe. A 3 wt.% alginate solution was prepared by dissolving SA powder in purified water through continuous magnetic stirring at 80 rpm for 2 h at 65 °C. To obtain obvious fiber images, red ink was mixed within SA. For cryogenic printing of scaffolds, a commercial 3D printer (MAM-II, Fochif, China) was modified by installing a specially designed cryogenic substrate. The cryogenic substrate temperature can be adjusted as low as −20 °C through a cooling pump (DLSB 10/80, Teer Equipment Co., Ltd., Zhengzhou, China). Two syringes loaded with PCL and alginate solutions were set with a coaxial printhead separately (1825, Changsha Nayi Instrument Technology Co., Ltd., China). The printing was carried out on −20 °C substrate to form a coaxial 3D porous scaffold (Figure 1). The pneumatic pressures were applied on these two printheads separately through different tunnels. The printing process of coaxial scaffolds was optimized by investigating the effect of different processing parameters, such as the air pressure of inner nozzle, the air pressure of outer nozzle, the concentration of PCL, and the concentration of alginate. The printed scaffolds, containing frozen GAC or water, were placed in a −20 °C freezer for 24 h and then air-dried under 20 °C for 36 h. At last, the scaffolds were lyophilized for 36 h to remove the residual solvent.

Meanwhile, groups of CP scaffolds were fabricated for comparison. A 40 wt.% PCL solution was extruded from the chamber through an 18G metal nozzle via pneumatic pressures and the printing process carried out on −20 °C substrate. The printed CP scaffolds were then immediately lyophilized for 36 h. In practical scenarios, different scaffolds were printed with varying parameters: for instance, scaffolds with dimensions of 24 × 24 mm^2^ and filament offsets of 800 μm, 1000 μm, and 1200 μm were printed and named CCP800, CCP1000, and CCP1200, respectively. For comparison, the CP scaffolds (named CP1000) with the same overall and inner structures of CCP1000 were fabricated. The height of all scaffolds was kept as 1 mm unless stated otherwise. During printing, the ambient temperature (25 °C) and humidity (40–45%) were kept constant to minimize the environmental effect. For example, moisture-caused frost may affect the printing process and needs to be prevented.

### 2.3. Morphology Characterization

The macro and micro morphologies of 3D-printed coaxial scaffolds were observed under optical microscope (B011, Supereyes, Shenzhen, China) and field-emission scanning electron microscope (FESEM, JSM7600F, JEOL Ltd., Tokyo, Japan), respectively.

### 2.4. Mechanical Properties

**Figure 2:**
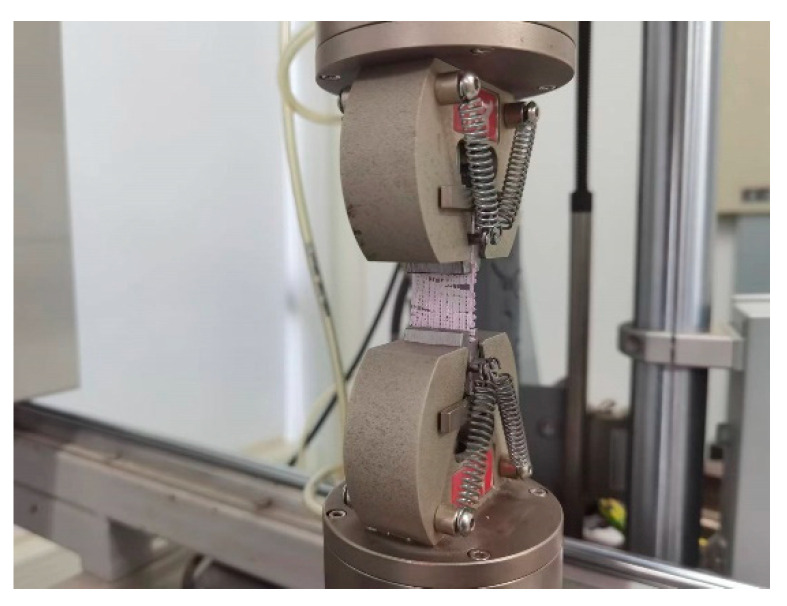
Photographs of the tensile performance test process.

The tensile test was performed on an electronic dynamic and static fatigue Testing Machine (E1000, ITW Group Instron, High Wycombe, UK) at the speed of 2 mm/min, as shown in Figure 2. The coaxial scaffolds of CCP 800, CCP 1000, and CCP 1200 groups and the cryogenic printed CP 1000 group were made in the shape of 24 mm long, 24 mm wide, and 1 mm thick. The engineering stress and strain were calculated using the following Equations (1) and (2):

Σ = F/(X∙Y),(1)

Δ = D/Z,(2)

where σ is the engineering stress of the coaxial scaffolds, δ is the engineering strain of the coaxial scaffolds, F is tensile force. X, Y, and Z were the pre-measured length, width, and height of coaxial scaffolds, respectively. The Young’s moduli were calculated from the linear regions of the engineering stress–strain curve.

### 2.5. Accelerated Acid Degradation

The coaxial scaffolds from CCP 1200, CCP 1000, CCP 800 groups, and the selected cryogenic scaffolds from CP 1000 group were immersed in 15 mL 5 M HCL aqueous solution and maintained at 37 °C to mimic the bacterial infection condition in vivo. Thereafter, the coaxial scaffolds were carefully rinsed with deionized water to remove the residual HCL until the pH became neutral, and then freeze-dried for 36 h. The dry weights of coaxial scaffolds were measured for comparison with the original state. The experiments were performed in triplicate for each group. The degradation rate was expressed as weight loss using the following Equation (3):

Weight Loss = (m_0_ − m)/m_0_ × 100%, (3)

where m_0_ and m represent the scaffold weights before and after acidic immersion, respectively.

### 2.6. In Vitro Release of Levofloxacin from Cryogenic Coaxial Scaffolds

To compare the drug release behavior of shell/core coaxial scaffold and solid scaffold, 2 wt.% levofloxacin was prepared as the core and physisorption, respectively. Polycaprolactone (PCL) and drug-loaded SA were made into cryogenic shell/core coaxial scaffolds. In addition, cryogenic PCL scaffolds were placed in an aqueous solution containing 2 wt.% levofloxacin for 1 h. After immersion. the PCL scaffolds were placed under −20 °C freezer, and then immediately lyophilized for 36 h to remove the residual solvent.

The coaxial scaffolds of CCP 800, CCP 1000, and CCP 1200 groups and the cryogenic scaffolds from CP 1000 group, made in the shape of 12 mm long, 12 mm wide, and 1 mm thick, were suspended in 10 mL of HCL (5 M). The accumulated amount of levofloxacin released in the solution was measured every hour and determined with a UV spectrophotometer (Lambda 35, American PerkinElmer, American) at *λ* = 300 nm.

### 2.7. Statistical Analysis

The data were presented as mean ± standard error (SE, *n* = 3) unless stated otherwise. Further, one-way analysis of variance and Tukey post hoc test were applied to evaluate the statistical significance.

## 3. Results and Discussion

### 3.1. Optimization of 3D Printing Process

The cryogenic coaxial printing is different from the traditional extrusion cryogenic printing and fused deposition modeling for polycaprolactone (PCL). This technology involves ultra-low temperature to shape the liquid filament immediately, and the extrusion of two materials.

**Figure 3:**
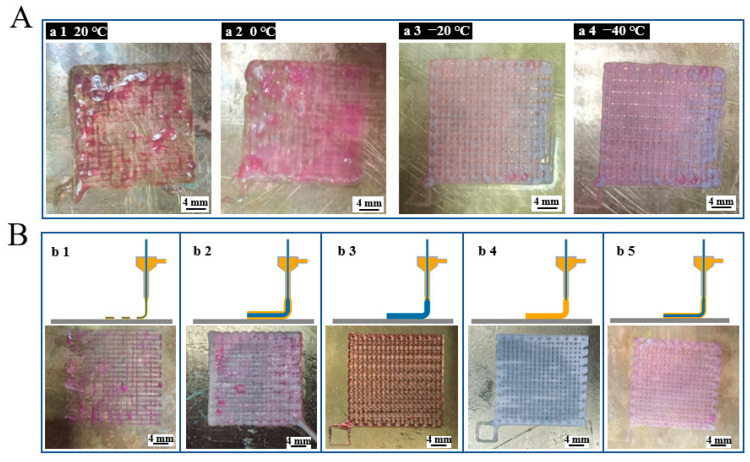
(**A**) Cryogenic coaxial printing under different temperatures; (**B**) cryogenic coaxial printing with different material feed speeds.

During printing, the temperature of the printing platform plays a vital role in the molding effect. If the molding temperature does not reach an appropriate value, the scaffold cannot be molded. Figure 3A(a1–a4) shows the molding conditions of the scaffolds when the substrate temperatures are 20 °C, 0 °C, −20 °C, and −40 °C, respectively. It can be seen that, when the temperature decreases, the outline of the scaffolds starts to be clear. The solidification temperature of glacial acetic acid is 16.6 °C, so the substrate temperature must be much lower than this temperature to stabilize the scaffold fiber formation. However, when the temperature decreases, the solidification speed of the PCL increases, generating the internal stress inside the fiber during the crystallization process. This leads to the warping of the scaffold. Besides, a too-low temperature will freeze the PCL and SA inside the coaxial nozzle, which halts the printing process. In summary, −20 °C was finally chosen to be the temperature of the printing substrate.

At the same time, the concentration of the inner and outer materials and the pertaining printing air pressures of the two materials will greatly affect the molding effect of the scaffold. Figure 3B(b1) depicts the discontinuity of the printed fibers when the printing air pressure is large or the material concentration is low. Figure 3B(b2) depicts the situation where the printed filament diameter is larger than the designed one and “swallows” the pores, leading to a nonporous scaffold. Figure 3B(b3) shows the situation where the inner SA diameter is too large to squeeze the outer PCL space when the inner SA concentration is too small or the inner pressure is high. Figure 3B(b4) describes the phenomenon where the outer PCL layer thickness is too large to narrow the inner SA when the outer PCL concentration is too small or the outer pressure is high. Figure 3B(b5) depicts the optimized coaxial scaffold morphology. The optical parameter set includes the SA concentration of 3%, the PCL concentration of 40%, the inner pressure between 120 and 170 MPa, and the outer pressure between 30 and 70 MPa.

### 3.2. Morphology of Scaffolds

**Figure 4:**
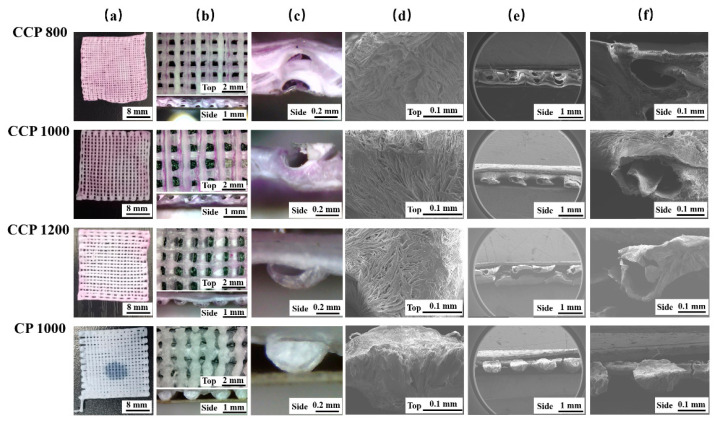
Morphology of CCP 800, CCP 1000, CCP 1200, and CP 1000 scaffolds. (**a**) Top view of overall scaffolds; (**b**) top and side views of an area of scaffolds; (**c**) the coaxial structure of fibers in scaffolds; (**d**) the micropores on printed fiber surface; (**e**) the side view of scaffolds; (**f**) the shell and core coaxial structure of the printed fiber of scaffolds.

Figure 4a,b describes the macroscopic appearance of the scaffolds. In the CP 1000 scaffold, the polycaprolactone (PCL) appears white under the optical microscope. In the characterized CCP 800, CCP 1000, and CCP 1200 scaffolds, the presence of sodium alginate mixed with red ink is the core in the coaxial scaffolds. In Figure 4c, the cross-sectional views of the scaffolds under higher magnification are shown. It can be seen in the pure solid fiber in the CP scaffold and the hollow tubular fiber in the CCP scaffolds. During printing, the forming of the scaffold filaments is mainly affected by the temperature difference between the ink extruded from the nozzle and the low-temperature substrate. The filament cross-section and the core of the filaments in Figure 4c are semicircular or elliptical. This is probably because the temperature decreasing speed of the depositing fiber is not fast enough, which causes the collapse of the lower part under the gravity of the upper part of one filament. However, with the solidification of PCL/GAC and SA, the whole fiber structure remains intact. Figure 4d shows the surface morphology of the four scaffolds. All of them have small and randomly distributed micropores. This is due to the directional growth of the GAC grains under the low-temperature field, and the filament temperature drops to the equilibrium value of dissolution [24]. Below the value, thermally induced phase separation occurs, leading to the precipitation of PCL and the separation and crystallization of PCL from GAC. These micropores’ morphologies on the fiber surfaces are reported helpful for cell adhesion, growth, and proliferation [25]. Figure 4e depicts the cross-section of the scaffold. It can be seen that the SA core does not fill in the entire coaxial inner cavity but shrinks to a thin string, thus forming a unique PCL–air–SA three-layer structure. That structure is different from previously reported cryogenic coaxial printing, in which the collagen shell contacts the PCL core closely without interspace [26]. The reason is that the inner layer of the SA is not freeze-dried directly; it is freeze-dried subsequent to natural air drying. This structure not only expands the surface area of the scaffolds but also increases the room that is conducive to cell adhesion and growth. It also forms a guided SA filamentous structure, which might be beneficial for nerve cell alignment. In summary, an obvious coaxial structure is observed in the CCP scaffold, which verified the success of cryogenic coaxial printing. Meanwhile, it retains the surface micropore characteristics of the CP scaffold. With the shrinkage of the SA core, the coaxial fiber manifests a special PCL–air–SA three-layer structure.

### 3.3. Tensile Properties of 3D-Printed Coaxial Scaffolds

**Figure 5:**
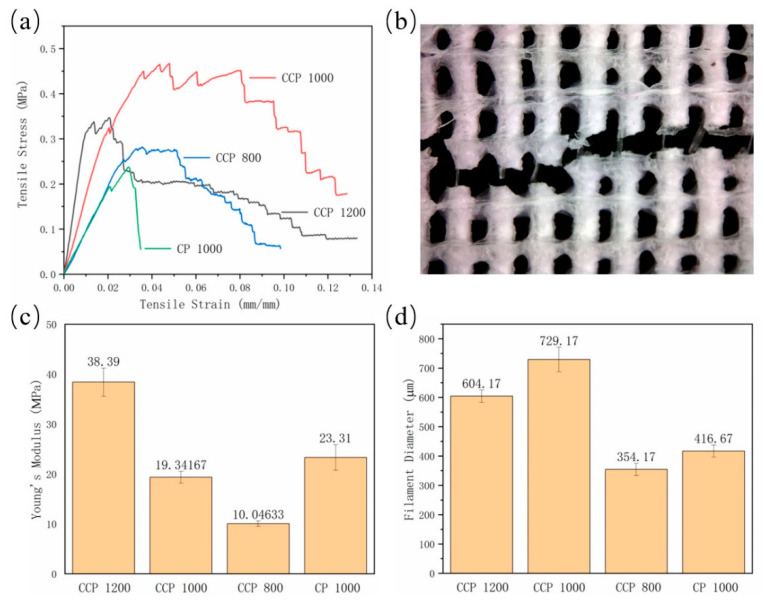
(**a**) The tensile stress/tensile strain curves of printed scaffolds; (**b**) the rupture of scaffold after tensile test; (**c**) the Young’s modulus of printed scaffolds; and (**d**) the filament diameter of printed scaffolds.

The tensile test was performed on the CP and CCP scaffolds, and the results are shown in Figure 5a. Specifically, the maximum tensile stress values of the CCP 1200, CCP 1000, CCP 800, and CP 1000 groups are 0.347 MPa, 0.463 MPa, 0.276 MPa, and 0.237 MPa, respectively. Among them, the CCP 1000 group has the highest tensile stress. When the side length of the whole scaffold is 24 mm × 24 mm, the smaller the filament offset is, the more filaments there will be in the scaffold. Theoretically, denser scaffold filament leads to higher tensile stress, but it is different from our results. We found that the filament offset will affect the temperature field, affecting the single filament-forming further. The forming of a filament will be affected by the temperature of the previous filament. The influence of the field results in a change in the filament diameter. The specific performance is shown in Figure 5d. The filament diameters of the CCP 1200, CCP 1000, CCP 800, and CP 1000 groups are 604.17 μm, 729.17 μm, 354.17 μm, and 416.67 μm, respectively. In the CCP 800 group, because the filament offset is too small, the low temperature of the previously formed filament could assist new filament formation rapidly, resulting in a smaller filament diameter. However, because the inner layer of the CP 1000 scaffold is not filled with sodium alginate, due to the sublimation of GAC in the post-processing process, the fibers in the CP 1000 scaffold are smaller than those of the CCP 1000 scaffold. This phenomenon is the same as our group’s previous research [27]. The maximum tensile stress of the scaffold is determined by the number of filaments in the scaffold, the strength of the filament, and the filament diameter. Although the number of filaments in the CCP 800 group is the largest, the maximum tensile stress is not high because the filament diameter is too thin. The CCP 1000 group has more filaments than the CCP 1200 group, and the single filament diameter is the largest, giving it the greatest tensile stress.

At the same time, we found that the tensile stress of the CCP 1200, CCP1000, and CCP 800 groups decreased stepwise and dropped to a stable value in the second half of stretching. After analyzing the tensile section of the scaffold, the main reason for the step-like decrease in the stress is the special coaxial structure. After the outer polycaprolactone (PCL) shell is stretched and broken, the inner sodium alginate undergoes a secondary process due to its better elasticity, supporting the scaffold until the PCL shell of the next fiber is broken by tension, and the whole tensile stress is further reduced. This process was repeated until all the filaments were broken, thereby forming a characteristic of a zig-zag period on the stress–strain curve. As shown in Figure 5b, we can see the core of sodium alginate string inside the ruptured PCL filament. In the scaffolds of the CP 1000 group, the tensile stress is shown as a straight break, which further verifies the coaxial structure of the CCP groups, and this kind of impact-tough scaffold has a strong application prospect in the damage of various membrane tissues [28].

In terms of the elastic properties, as shown in Figure 5c, the Young’s modulus values of the CCP 1200, CCP 1000, CCP 800, and CP 1000 groups are 38.39 MPa, 19.342 MPa, 10.046 MPa, and 23.32 MPa, respectively. Due to the addition of the inner layer of sodium alginate, the Young’s modulus of the CP 1000 is higher than that of the CP 800. If the fiber offset becomes smaller, the number of wires in the scaffold increases and the elastic modulus tends to decrease. In summary, the CCP scaffold has a higher maximum tensile stress and elastic modulus than the CP scaffold, and the unique coaxial structure enables it to absorb impact.

### 3.4. Accelerated Acidic Degradation

**Figure 6:**
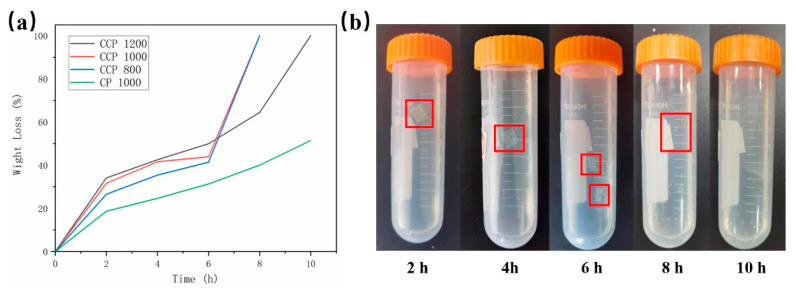
(**a**) Weight loss curve of printed scaffold in accelerated acidic degradation experiment; (**b**) degradation process of CCP 1000 scaffolds.

The results of the weight losses are shown in Figure 6a and Table 1. During the experiment, the CCP 1000 and CCP 800 scaffolds degraded 8 h later. The CCP 1200 scaffolds degraded after 10 h; meanwhile, the CP 1000 scaffolds showed 51.43 ± 1.27% of degradation. SA, as a hydrogel, degraded more rapidly than PCL in HCl. This led to the rate of weight loss in the CCP groups being faster than that in the CP group at the beginning. Over a period of 4 to 6 h, the rate of weight loss in the printed scaffolds tends to be consistent. This phenomenon illustrated that the SA in the CCP groups was all degraded and the PCL in the printed scaffolds was degrading at an approximate rate. Six hours after the experiment was performed, the degradation rate of the CCP groups was again faster than that of the CP group. The CCP scaffolds could not maintain the morphology after 6 h. This led to the PCL–air–SA structure being more exposed to HCl. The following higher porosity and more contact with HCl contributed to this phenomenon. As shown in Figure 6a, the degradation time of the CCP 1200 scaffolds was longer than that of the CCP 1000 and CCP 800 groups. Presumably, the CCP 1200 scaffolds have higher elastic modulus than the printed scaffolds. This allowed them to maintain their morphology in HCl for a longer time and exposed their internal structure to HCl less early so as to obtain a longer degradation time.

As shown in Figure 6b, in the degradation solution, the morphology of the CCP1000 scaffolds was completely clear from the beginning 2 h, and, by 4 h, the scaffolds still maintained the morphology but showed cracks in the surface. After 6 h, the scaffolds fractured into two parts and could not maintain the normal scaffold morphology. The scaffolds decomposed to filaments after 8 h, at which time they were considered completely degraded. Almost no scaffolds remained in the degradation solution after 10 h.

### 3.5. Drug-Sustained Release Properties of Coaxial Scaffolds

**Figure 7:**
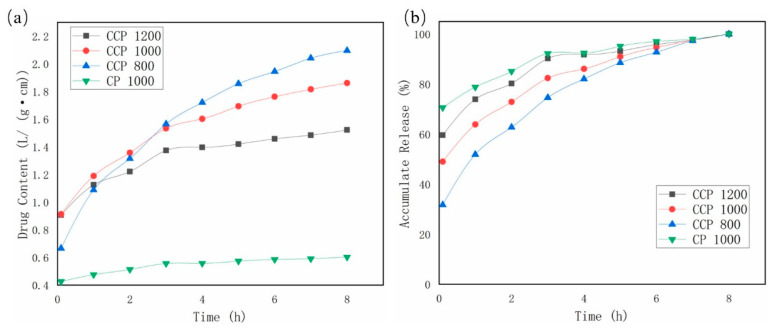
(**a**) Drug release curve of printed scaffolds; (**b**) drug accumulation curve of printed scaffolds.

Figure 7a depicts the drug release content in the scaffold as a function of time. It finds that, with the prolongation of time, the drug in the scaffold is gradually released, and the amount of drug in the solution increases gradually. After 8 h, the drug content was the highest in the CCP 800 group, followed by the CCP 1000 group, third in the CCP 1200 group, and the lowest in the CP 1000 group. Further, the drug content of the CP 1000 group was significantly lower than that of the other three groups. It is demonstrated that, by blending the drug with the hydrogel and using the coaxial printing process, the scaffold can carry more drug, which is more conducive to the recovery of damaged tissue because, under the same external dimensions, the smaller the fiber interval is, the greater the fiber number there will be, and the number of fibers determines the drug content of the coaxial scaffold directly. The larger the number of fibers, the higher the drug content, so the CCP 800 group has the largest drug content, and the CCP 1200 group has the smallest content.

Figure 7b shows the release rate of the drug increased with time in each group. It can be seen from the figure that, at the beginning of the release, more than 70% of the drugs in the CP 1000 group were released. In contrast, the release amounts of CCP 1200, CCP 1000, and CCP 800 are significantly smaller, and it can be clearly found that the drug release of the CP 1000 group reached more than 90% after 3 h, while the drug of the CCP group has a longer release time, which verifies that the scaffold has a sustained release function. Filament offset also has an effect on the release of levofloxacin. Due to the limited solubility of levofloxacin in hydrochloric acid, the local concentration of levofloxacin around a single filament will be saturated, thereby inhibiting the release of levofloxacin. It is further shown that, the smaller the filament offset is, the longer the drug release time is, and the stronger the drug-sustained release ability is.

## 4. Conclusions

In this study, firstly, a PCL/SA coaxial scaffold with a porous structure was fabricated through cryogenic coaxial 3D printing, successfully proving the fabrication feasibility of a 3D coaxial scaffold with a polymeric shell. Secondly, the obtained scaffold possesses both a porous outer shell and a string-like core. This core string–sheath tube structure can be used as a nerve guide conduit (NGC), which might be beneficial to guide nerve cells’ growth. As a result, the outer porous structure assists bone repair and the inner structure assists nerve repair. Thirdly, the cryogenic coaxial scaffolds have improved maximum tensile stress and elastic modulus and the capability of loading more drug compared to cryogenic printed scaffolds. In addition, the coaxial scaffolds possess a “responsive” drug release capability to an acidic environment. At last, cryogenic coaxial printing is a multi-physical process involving thermal, fluid fields, and dual material phases. This study elicits potential results and broadens the research field.

## Figures and Tables

**Table 1:** Weight loss results of printed scaffold in accelerated acidic degradation experiment.

	Samples	CCP 1200	CCP 1000	CCP 800	CP 1000
Time (h)					
2		34.05% ± 8.64%	31.55% ± 2.14%	26.51% ± 0.08%	18.60% ± 5.48%
4		42.49% ± 3.00%	41.54% ± 11.47%	35.38% ± 9.73%	21.58% ± 6.27%
6		49.93% ± 7.70%	43.88% ± 9.52%	41.39% ± 2.33%	31.26% ± 4.71%
8		64.43% ± 6.92%	100%	100%	40.02% ± 1.11%
10		100%	100%	100%	51.43% ± 1.27%

## Data Availability

The raw/processed data required to reproduce these findings cannot be shared at this time due to technical or time limitations.

## References

[1] Vanaei H.R., Khelladi S., Deligant M., Shirinbayan M., Tcharkhtchi A. (2022). Numerical Prediction for Temperature Profile of Parts Manufactured using Fused Filament Fabrication. J. Manuf. Process..

[2] Yang B., Liu T., Gao G., Zhang X., Wu B. (2022). Fabrication of 3D GelMA Scaffolds Using Agarose Microgel Embedded Printing. Micromachines.

[3] Li S., Wang K., Jiang X., Hu Q., Zhang C., Wang B. (2020). Rapid Fabrication of Ready-to-Use Gelatin Scaffolds with Prevascular Networks Using Alginate Hollow Fibers as Sacrificial Templates. ACS Biomater. Sci. Eng..

[4] Paredes C., Martínez-Vázquez F.J., Pajares A., Miranda P. (2019). Development by robocasting and mechanical characterization of hybrid HA/PCL coaxial scaffolds for biomedical applications. J. Eur. Ceram. Soc..

[5] Zhang W., Feng C., Yang G., Li G., Ding X., Wang S., Dou Y., Zhang Z., Chang J., Wu C. (2017). 3D-printed scaffolds with synergistic effect of hollow-pipe structure and bioactive ions for vascularized bone regeneration. Biomaterials.

[6] Singh N.K., Han W., Nam S.A., Kim J.W., Kim J.Y., Kim Y.K., Cho D.W. (2020). Three-dimensional cell-printing of advanced renal tubular tissue analogue. Biomaterials.

[7] Zhou X., Nowicki M., Sun H., Hann S.Y., Cui H., Esworthy T., Lee J.D., Plesniak M., Zhang L.G. (2020). 3D Bioprinting-Tunable Small-Diameter Blood Vessels with Biomimetic Biphasic Cell Layers. ACS Appl. Mater. Interfaces.

[8] Chuysinuan P., Pengsuk C., Lirdprapamongkol K., Techasakul S., Svasti J., Nooeaid P. (2019). Enhanced Structural Stability and Controlled Drug Release of Hydrophilic Antibiotic-Loaded Alginate/Soy Protein Isolate Core-Sheath Fibers for Tissue Engineering Applications. Fibers Polym..

[9] Yuan H., Li X., Lee M.S., Zhang Z., Li B., Xuan H., Li W.J., Zhang Y. (2021). Collagen and chondroitin sulfate functionalized bioinspired fibers for tendon tissue engineering application. Int. J. Biol. Macromol..

[10] Sang Q., Li H., Williams G., Wu H., Zhu L.M. (2018). Core-shell poly(lactide-co-ε-caprolactone)-gelatin fiber scaffolds as pH-sensitive drug delivery systems. J. Biomater. Appl..

[11] Yi B., Shen Y., Tang H., Wang X., Li B., Zhang Y. (2019). Stiffness of Aligned Fibers Regulates the Phenotypic Expression of Vascular Smooth Muscle Cells. ACS Appl. Mater. Interfaces.

[12] Mousavi Nejad Z., Zamanian A., Saeidifar M., Vanaei H.R., Amoli M.S. (2021). 3D Bioprinting of Polycaprolactone-Based Scaffolds for Pulp-Dentin Regeneration: Investigation of Physicochemical and Biological Behavior. Polymers.

[13] Pi Q., Maharjan S., Yan X., Liu X., Singh B., van Genderen A.M., Robledo-Padilla F., Parra-Saldivar R., Hu N., Jia W. (2018). Digitally Tunable Microfluidic Bioprinting of Multilayered Cannular Tissues. Adv. Mater..

[14] Bagnol R., Sprecher C., Peroglio M., Chevalier J., Mahou R., Büchler P., Richards G., Eglin D. (2021). Coaxial micro-extrusion of a calcium phosphate ink with aqueous solvents improves printing stability, structure fidelity and mechanical properties. Acta Biomater..

[15] Cornock R., Beirne S., Thompson B., Wallace G.G. (2014). Coaxial additive manufacture of biomaterial composite scaffolds for tissue engineering. Biofabrication.

[16] Hu Y., Wu B., Xiong Y., Tao R., Panayi A.C., Chen L., Tian W., Xue H., Shi L., Zhang X. (2021). Cryogenic 3D printed hydrogel scaffolds loading exosomes accelerate diabetic wound healing. Chem. Eng. J..

[17] Sadowska J.M., Genoud K.J., Kelly D.J., O'Brien F.J. (2021). Bone biomaterials for overcoming antimicrobial resistance: Advances in non-antibiotic antimicrobial approaches for regeneration of infected osseous tissue. Mater. Today.

[18] Wright J.A., Nair S.P. (2010). Interaction of staphylococci with bone. Int. J. Med. Microbiol..

[19] Montoya C., Du Y., Gianforcaro A.L., Orrego S., Yang M., Lelkes P.I. (2021). On the road to smart biomaterials for bone research: Definitions, concepts, advances, and outlook. Bone Res..

[20] Xiong M.H., Bao Y., Yang X.Z., Wang Y.C., Sun B., Wang J. (2012). Lipase-sensitive polymeric triple-layered nanogel for “on-demand” drug delivery. J. Am. Chem. Soc..

[21] Li L.L., Xu J.H., Qi G.B., Zhao X., Yu F., Wang H. (2014). Core-shell supramolecular gelatin nanoparticles for adaptive and “on-demand” antibiotic delivery. ACS Nano.

[22] Chu L., Gao H., Cheng T., Zhang Y., Liu J., Huang F., Yang C., Shi L., Liu J. (2016). A charge-adaptive nanosystem for prolonged and enhanced: In vivo antibiotic delivery. Chem. Commun..

[23] Won J.Y., Kim J., Gao G., Kim J., Jang J., Park Y.H., Cho D.W. (2020). 3D printing of drug-loaded multi-shell rods for local delivery of bevacizumab and dexamethasone: A synergetic therapy for retinal vascular diseases. Acta Biomater..

[24] van de Witte P., Dijkstra P.J., van den Berg J.W.A., Feijen J. (1996). Phase separation processes in polymer solutions in relation to membrane formation. J. Membr. Sci..

[25] Sun T., Meng C., Ding Q., Yu K., Zhang X., Zhang W., Tian W., Zhang Q., Guo X., Wu B. (2021). In situ bone regeneration with sequential delivery of aptamer and BMP2 from an ECM-based scaffold fabricated by cryogenic free-form extrusion. Bioact. Mater..

[26] Kim G., Ahn S., Kim Y., Cho Y., Chun W. (2011). Coaxial structured collagen-alginate scaffolds: Fabrication, physical properties, and biomedical application for skin tissue regeneration. J. Mater. Chem..

[27] Zhang W., Ullah I., Shi L., Zhang Y., Ou H., Zhou J., Ullah M.W., Zhang X., Li W. (2019). Fabrication and characterization of porous polycaprolactone scaffold via extrusion-based cryogenic 3D printing for tissue engineering. Mater. Des..

[28] Pitaluga L.H., Souza M.T., Zanotto E.D., Romero M.E.S., Hatton P.V. (2018). Electrospun F18 Bioactive Glass/PCL-Poly (epsilon-caprolactone)-Membrane for Guided Tissue Regeneration. Materials.

